# Incubation temperature, morphology and performance in loggerhead (*Caretta caretta*) turtle hatchlings from Mon Repos, Queensland, Australia

**DOI:** 10.1242/bio.20148995

**Published:** 2015-05-22

**Authors:** Elizabeth L. Sim, David T. Booth, Colin J. Limpus

**Affiliations:** 1 School of Biological Sciences, The University of Queensland, Brisbane, QLD 4072, Australia; 2 Department of Environment and Heritage Protection, PO Box 2454, Brisbane, QLD 4001, Australia

**Keywords:** Loggerhead turtles, Body size, Nest, Swimming performance, Crawling speed, Self-righting ability

## Abstract

Marine turtles are vulnerable to climate change because their life history and reproduction are tied to environmental temperatures. The egg incubation stage is arguably the most vulnerable stage, because marine turtle eggs require a narrow range of temperatures for successful incubation. Additionally, incubation temperature affects sex, emergence success, morphology and locomotor performance of hatchlings. Hatchlings often experience high rates of predation in the first few hours of their life, and increased size or locomotor ability may improve their chances of survival. Between 2010 and 2013 we monitored the temperature of loggerhead (*Caretta caretta*; Linnaeus 1758) turtle nests at Mon Repos Rookery, and used these data to calculate a mean three day maximum temperature (T3dm) for each nest. We calculated the hatching and emergence success for each nest, then measured the mass, size and locomotor performance of hatchlings that emerged from those nests. Nests with a T3dm greater than 34°C experienced a lower emergence success and produced smaller hatchlings than nests with a T3dm lower than 34°C. Hatchlings from nests with a T3dm below 34°C performed better in crawling and swimming trials than hatchlings from nests with a T3dm above 34°C. Thus even non-lethal increases in global temperatures have the potential to detrimentally affect fitness and survival of marine turtle hatchlings.

## INTRODUCTION

Many marine turtle populations have experienced drastic population declines over the past thirty years due to anthropogenic disturbances, habitat loss, marine debris and predation (Lutcavage et al., 1997; Hamann et al., 2007; Wallace et al., 2011). With impending rises in global temperatures (IPCC, 2007), marine turtles are likely to face threats to many stages of their life cycle. The oceanic currents that drive hatchling dispersal may become disrupted (Hamann et al., 2011; Van Houtan and Halley, 2011), food availability may be affected (Hawkes et al., 2009) and shifts in latitude may occur as a response to oceanic warming (Witt et al., 2010). Nesting grounds face threats such as sea level rise (Fish et al., 2005; Hamann et al., 2007), increased cyclonic activity (Poloczanska et al., 2009; Fuentes and Abbs, 2010) and increased nest temperatures (Booth et al., 2004; Glen and Mrosovsky, 2004; Hawkes et al., 2007; Fuentes et al., 2010; Booth and Evans, 2011; Booth et al., 2013).

Nest temperature is determined by a combination of sand temperature and metabolic heating produced by developing embryos (Broderick et al., 2001; Booth and Freeman, 2006). Metabolic heat can raise the nest temperature to 2–6°C above sand temperature in the final weeks of incubation (Broderick et al., 2001; van de Merwe et al., 2006; Zbinden et al., 2006). Constant incubation temperature experiments performed in the laboratory have shown that marine turtle eggs fail to hatch when incubated below 24°C or above 34°C (Blanck and Sawyer, 1981; Miller, 1985; Ackerman, 1997; Matsuzawa et al., 2002; Carthy et al., 2003; Bustard, 1971; Bustard and Greenham, 1968). However, field-based studies have shown that unhatched embryos can survive temperatures above 34°C for short periods of time, particularly during later stages of development (Maulany et al., 2012a; Maulany et al., 2012b; Booth et al., 2013; Ewert, 1979; Limpus et al., 1985; Miller, 1985).

Even sub-lethal incubation temperatures can affect emergence, morphology and locomotor performance of marine turtle hatchlings (Reece et al., 2002; Glen and Mrosovsky, 2004; Burgess et al., 2006; Ischer et al., 2009; Booth and Evans, 2011). Nests with higher incubation temperatures produce smaller hatchlings with a larger residual yolk (Reece et al., 2002; Booth, 2006; Burgess et al., 2006), which are often more susceptible to predation by gape-limited predators (Bustard, 1972; Gyuris, 2000; Burgess et al., 2006). The risk of predation is highest as hatchlings crawl down the beach and swim across the shallow reef (Gyuris, 1994; Davenport, 1997). Hatchlings generally do not actively avoid or defend themselves against predators (Gyuris, 1994), which means hatchlings that can move more quickly through this “gauntlet” of predators may have a greater chance of surviving the initial few hours.

Most studies of the effects of incubation temperature on hatchling performance have used either controlled constant temperature incubation in the laboratory or the mean incubation temperature for naturally-incubated nests on beaches. Recently some studies have shown that the mean three day maximum temperature (T3dm) may be a more accurate predictor of hatchling performance in olive ridley turtles (*Lepidchelys olivacea*) (Maulany et al., 2012a) than mean temperature. Despite having a mean temperature in the normal range, nests with a T3dm above 34°C had a lower emergence success, and these nests produced hatchlings that performed worse in locomotor performance trials (Maulany et al., 2012a). That study was conducted in Indonesia, which is a tropical environment, and accordingly has high sand temperatures and high rainfall (Maulany et al., 2012b). Studies in sub-tropical environments have not been conducted, nor have studies on other marine turtle species. With impending climate change, it is important to understand how increasing temperatures will affect sea turtle nests worldwide.

In this paper we investigate the effect of T3dm on emergence success hatchling size and hatchling locomotor performance using the loggerhead turtle (*Caretta caretta*) at a sub-tropical rookery in Queensland, Australia. We predicted that nests with a T3dm above 34°C would have a lower emergence success, and produce hatchlings that are smaller and exhibit decreased locomotor performance compared to hatchlings from nests with a T3dm below 34°C.

## MATERIALS AND METHODS

### Study site

We conducted this study at Mon Repos Conservation Park (24°48′S, 152°27′E) in south east Queensland, Australia. Mon Repos is a major sub-tropical *C. caretta* rookery with between 100 and 600 females nesting each year (Limpus, 2009). All research was carried out in accordance with Animal Ethics Approval Certificate SBS/316/10 from the University of Queensland.

### Collection of eggs

In December 2010, 2011 and 2012 we located nesting female *C. caretta* by patrolling the beach between dusk and dawn. We opportunistically and randomly selected 44 clutches of eggs laid by 37 females (25 in 2010, 12 in 2011 and 7 in 2012). We counted the eggs in each clutch and randomly selected a sample of 10 eggs, which we then weighed (±0.1 g) using an electronic balance (model EK-1200A, A&D, Tokyo, Japan). We relocated the clutches to a hatchery area located in the dunes of Mon Repos beach within two hours of oviposition. To generate a range of incubation temperatures we placed these relocated clutches either in full sun, half shade or full shade, to simulate shade conditions of dune vegetation on natural nests. We calibrated temperature data loggers (ibutton model DS1922L, Maxim Integrated, San Jose, CA, USA) in a water bath with a certified thermometer and determined they were accurate to ±0.2°C. We then set them to record the temperature every two (at a resolution of 0.0625°C) hours and placed one in the centre of each nest.

### Hatchling collection

After each nest had been incubating for 50 days, we placed a plastic enclosure around the top of the nest at dusk. We checked these enclosures every half hour between dusk and dawn to ensure that hatchlings were not on the surface long before being tested for locomotor performance. As soon as we discovered an emerging nest, we randomly selected between eight and sixty hatchlings, depending on how many there were in the first emergence. We transported the hatchlings to the laboratory in a 10 L bucket. We collected a total of 1653 hatchlings.

### Hatchling measurements

Once in the laboratory, we weighed each hatchling (±0.1 g) with an electronic balance (model EK-1200A, A&D, Tokyo, Japan). We then measured the straight carapace length and width at the widest point (±0.1 mm) with digital callipers (model 06915, Sontax, Perth, WA, Australia). We calculated carapace size index by multiplying length by width to give a value in mm^2^.

### Self-righting experiments

We began locomotor experiments within an hour of first collecting emergent hatchlings. To quantify righting performance we used the same method as Booth et al. (Booth et al., 2013). We placed each hatchling upside down on its carapace on a flat area of sand and timed how long it took to self-right. If a hatchling failed to self-right within 10 s, we returned it to its plastron for 10 s before the next trial. We repeated this procedure until the hatchling had successfully self-righted three times, or had attempted self-righting six times, whichever came first. We then assigned the hatchling a righting propensity score from 0 (failed to self-right) to 6 (successfully self-righted three trials out of three attempts) (Booth et al., 2013).

### Crawling ability

In order to control for the effect of body temperature on performance in ectotherms, we measured the plastron surface temperature of each hatchling with an infra-red thermometer (model AR300, Smart Sensor, Houston, TX, USA). We then measured hatchling crawling speed using the same method as Ischer et al. (Ischer et al., 2009). We placed each hatchling at the landward end of a 2.9 m length of black plastic guttering lined with moist, lightly compacted beach sand. The runway was 10 cm wide and contained a dim light at the seaward end to attract the hatchling and ensure that it crawled in a straight line. We timed each hatchling crawling along the guttering with a stopwatch and converted this value to cm/s.

### Swimming ability

Immediately following crawling trials we selected eight hatchlings from each sample. We measured swimming ability using the same method as Ischer et al. (Ischer et al., 2009), by fitting hatchlings with a Lycra harness that contained a monofilament line and was attached to a force transducer (model MLT050, ADInstruments, Sydney, NSW, Australia). This was connected to a bridge amplifier (model ML112 ADInstruments, Sydney, NSW, Australia) and the output was recorded via a data acquisition system (PowerLab model 8/20, ADInstruments, Sydney, NSW, Australia) programmed to sample force 40 times per second. Hatchlings swam in plastic tubs containing sea water maintained at 28°C for four hours. We calibrated the transducers before and after each trial by hanging a known mass from each one. We quantified swimming performance for each individual hatchling turtle by using LabChart v7.0 to calculate mean thrust (mN), stroke rate, proportion of time spent power-stroking and mean thrust per power stroke for each 10-min period throughout the 4 hour swimming trial, as per Pereira et al. (Pereira et al., 2011).

### Nest excavations

Between two and five days after the emergence of the first group of hatchlings, we excavated each nest and retrieved the data logger. We counted the number of hatched and unhatched eggs found in the nest and determined the hatching success and emergence success. We downloaded the data and calculated the mean temperature for the three warmest days of incubation (T3dm). We then split the nests into two groups, those with a T3dm below 34°C, and those with a T3dm above 34°C, as used by Maulany et al. (Maulany et al., 2012a).

### Statistics

We averaged all variables across hatchlings sampled to get a mean value for each nest. We used an ANOVA to test for a difference between the T3dm above and below 34°C groups. Specifically, to test the effect of T3dm above or below 34°C on hatching success and emergence success we used a nested ANOVA with clutch nested within maternal identity as a random factor. To test the effect of T3dm on hatchling mass, carapace length, carapace width and carapace size index, we used a nested ANCOVA with egg mass as a covariate and clutch nested within maternal identity as a random factor. Finally, to test for differences in righting ability or crawling speed, we used a nested ANCOVA with nests as the data unit, plastron surface temperature as a covariate and clutch nested within maternal identity as a random factor. We removed non-significant interactions. If there was a significant difference we added carapace size index to the model as a covariate to determine whether these differences were solely due to a difference in hatchling size. To test for the effect of T3dm on all swimming attributes, we used a repeated measures ANOVA.

We performed data analysis using R (R Development Core Team, version 2.15.0, 2013) and Statistica (Version 12). Data are reported as means and standard errors of means or as least squares covariate means and we assumed statistical significance if p<0.05.

## RESULTS

**Fig. 1.**
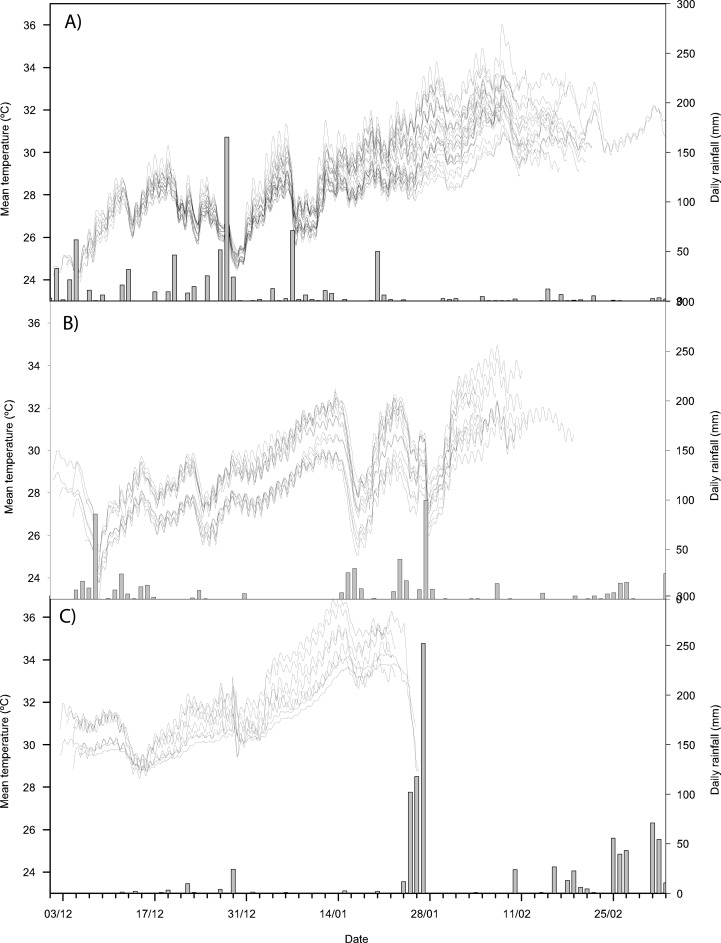
Daily rainfall (bars) and temperature profiles (lines) experienced by *C. caretta* nests in (A) 2010–2011 (n = 25), (B) 2011–2012 (n = 12) and (C) 2012–2013 (n = 7). Note that nests in the 2010–2011 and 2011–2012 seasons were obtained over several weeks, whereas nests in the 2012–2013 season were all obtained in the same week.

Mean nest temperatures were similar in the 2010–11 and 2011–12 seasons (mean (± SE) of 29.0±0.1°C (range of 27.9–30.9°C) in 2010–11 and 29.1±0.2°C (range of 27.9–30.9°C) in 2011–12; Fig. 1), but the 2012–13 season was warmer (mean of 31.6±0.2°C (range of 31.0–32.6°C); F_1,60_ = 34.05, p<0.001; Fig. 1). Nests in the shaded hatchery were approximately 1°C cooler than nests on the open beach (t_1,49_ = 8.44, p<0.001). Nest temperatures decreased after heavy rainfall events in all three seasons (Fig. 1). There were nests with a T3dm above and below 34°C in all years (Table 1).

**Table 1.**
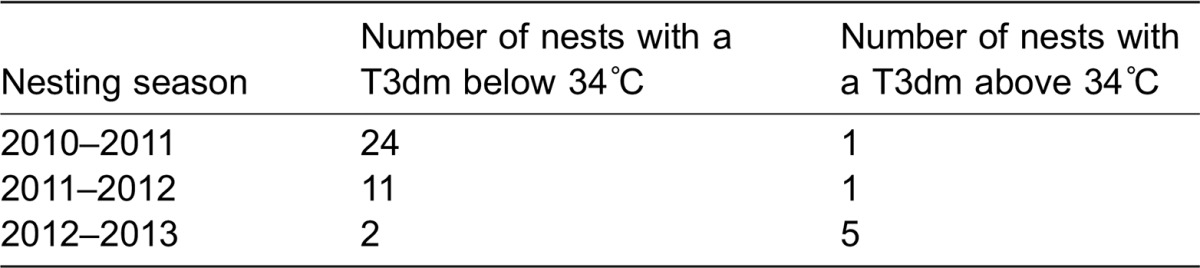
Number of *C. caretta* nests with a mean three day maximum temperature below 34°C and above 34°C in the 2010–2011, 2011–2012 and 2012–2013 nesting seasons

The mean incubation temperature was higher in the T3dm >34°C nests than the T3dm <34°C nests (Table 2). Nests with a T3dm >34°C had a lower emergence success than nests with a T3dm <34°C, but there was no difference in hatch success (Table 2). There was no difference in body mass or carapace length between hatchlings from the T3dm <34°C and T3dm >34°C nests (Table 2). There was no correlation between the clutch size and T3dm (F_1,60_ = 0.29, p = 0.60, R^2^ = 0.005).

**Table 2.**
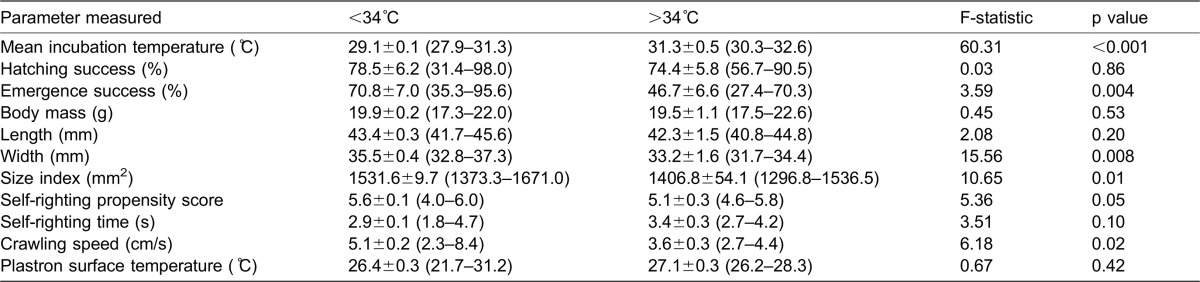
Mean ± standard error and range for incubation temperature, emergence and hatching success, hatchling morphological parameters, and hatchling locomotor performance of *C. caretta* nests with a T3dm (mean 3 day maximum temperature) below 34°C (n = 37) and above 34°C (n = 7)

Hatchlings from the T3dm <34°C nests had a wider carapace than hatchlings from the T3dm >34°C nests and consequently a larger carapace size index (Table 2). This difference in size did not translate into a difference in either self-righting propensity or time taken to self-right (Table 2). However hatchlings from the T3dm <34°C nests were faster crawlers than hatchlings from the T3dm >34°C nests (Table 2). When we added size index as a covariate, the difference between the groups remained (F_1,19_ = 6.56, p = 0.02). When we plotted these four significant variables continuously, there was a clear decline.

**Fig. 2.**
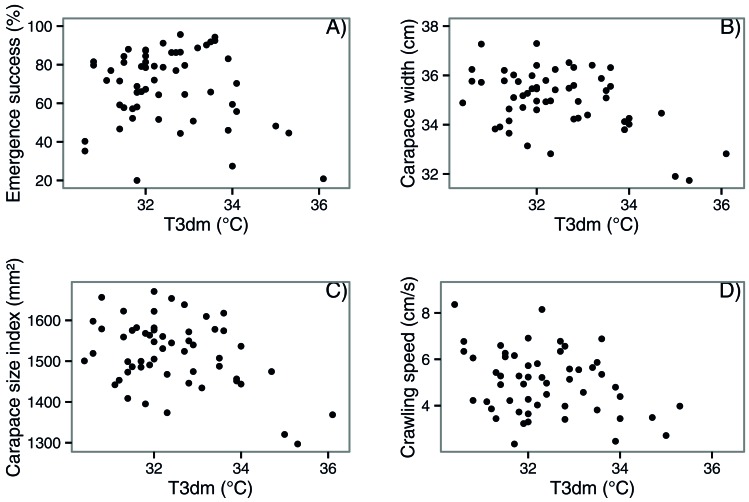
Emergence success (A), mean carapace width (B), mean carapace size index (C) and mean crawling speed (D) of *C. caretta* hatchlings plotted against the T3dm (mean maximum 3 day temperature)

For individual hatchlings, plastron surface temperature was negatively correlated with mean time taken to self-right (equation: SRT = 4.69−0.06*PST, F_1,1609_ = 16.51, p<0.001, R^2^ = 0.009) and positively correlated with crawling speed (equation: CS = 0.08*PST+3.04; F_1,1589_ = 13.26; p<0.001, R^2^ = 0.007). There was no correlation between plastron surface temperature and self-righting propensity (F_1,1621_ = 2.85, p = 0.09). When we plotted the data continuously, there was a clear decline when the T3dm was above 34°C (Fig. 2)

**Fig. 3.**
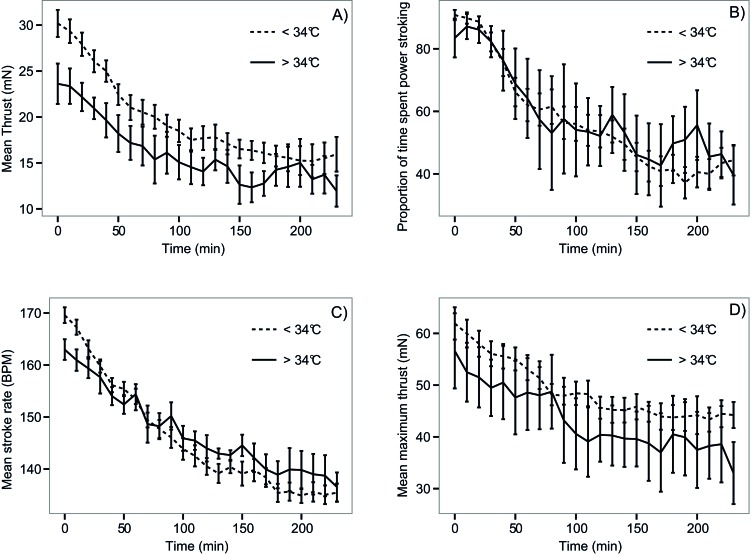
Mean thrust produced (A), proportion of time spent power stroking (B), mean stroke rate (C) and mean maximum thrust (D) of *C. caretta* hatchlings from nests with a T3dm (mean maximum 3 day temperature) below 34°C and above 34°C. Data were measured over a four hour period. Error bars indicate standard error.

Swimming parameters decreased as time spent swimming increased in both temperature categories (Fig. 3). Hatchlings from nests with a T3dm <34°C produced more thrust than hatchlings from nests with a T3dm >34°C for the first twenty minutes of swimming (p = 0.03–0.04; Fig. 3). There was no difference in stroke rate, proportion of time spent power stroking or mean maximum thrust between the two groups (Fig. 3).

## DISCUSSION

### Nest temperature

Mean nest temperatures recorded in this study fell within the range observed in this rookery in the recent past (30.0–33.1°C in the 2005–06 season and 28.9–32.7°C in the 2006–07 season (Chu et al., 2008), 28.9–32.7°C in the 2009–10 season (Wood et al., 2014) and 28.1–30.6°C in the 2010–11 season (Read et al., 2012)). Nest temperatures followed a similar pattern across years, becoming warmer towards the end of incubation due to metabolic heating (Zbinden et al., 2006). Thus, the T3dm was usually towards the end of the incubation period when the hatchlings were well developed. Clutch size did not correlate with T3dm, suggesting metabolic heating affected all nests in a similar way, and did not contribute to thermal differences between nests.

Several environmental factors appeared to affect nest temperature in this study. Nests in the shaded hatchery were 1°C cooler on average than nests on the open beach, and most nests experienced a drop in temperature following heavy rainfall. These environmental effects have been shown in other studies (Wood et al., 2014; Houghton et al., 2007), suggesting that the effects of climate change are not limited to increased temperatures. While all the nests in this study were relocated, it is unlikely that relocation affected nest temperatures, since nests were relocated to the same beach (DeGregorio and Williard, 2011; Pintus et al., 2009; Tuttle and Rostal, 2010).

### Hatching and emergence success

As we did not find an effect of T3dm on hatchling success, it is likely that temperatures in this study were not consistently high enough to negatively affect hatching success, as this has been shown in constant incubation temperature experiments in the laboratory, which used higher temperatures (Bustard and Greenham, 1968; Bustard, 1971) Other studies with a similar temperature range have also shown no correlation between temperature and hatching success (Chu et al., 2008; Hewavisenthi and Parmenter, 2002). However, similar mean nest temperatures have been correlated with a reduction in emergence success in other *C. caretta* nests (Chu et al., 2008; Read et al., 2012). More specifically, nests which experienced high temperatures in the last few days of incubation experienced a lower emergence success than those which did not (Maulany et al., 2012b; Matsuzawa et al., 2002).

The effect of T3dm on emergence success but not hatching success in this study suggests that increased nest temperatures are affecting hatchlings directly, either by the heat causing death (Matsuzawa et al., 2002) or decreasing hatchling activity and preventing the hatchlings from emerging (Mrosovsky, 1968; Drake and Spotila, 2002). Critical thermal maxima for other sea turtle species have been measured at between 37.1°C and 41.4°C (Drake and Spotila, 2002), which is higher than the sand temperatures measured in this study, however hatchlings began to display uncoordinated movements at 33.4°C (Drake and Spotila, 2002). Similarly critical threshold surface temperatures (above which hatchlings will not emerge) of between 32.4°C and has been calculated for *C. caretta* hatchlings in Florida (Moran et al., 1999). It is likely above this temperature coordinated muscle movement is inhibited (Matsuzawa et al., 2002).

Although all nests in this study were relocated, it is unlikely that this relocation affected the emergence or hatching success independently of temperature. Other studies have found no difference between relocated and *in situ* nests (Kornaraki et al., 2006; Wyneken et al., 1988; Tuttle and Rostal, 2010). One study has shown that hatching success is lower in relocated nests than in *in situ* nests (Pintus et al., 2009), however these eggs were relocated up to six hours after oviposition, so they may have been subject to movement-induced mortality (Limpus et al., 1979). All clutches in this study were relocated within two hours of oviposition.

One of the limitations of this study was the smaller number of nests with a T3dm above 34°C compared to nests with a T3dm below 34°C, and the fact that these were mostly concentrated in one season. This arose from our decision to incubate nests on a beach with variable thermal conditions, rather than control conditions in a laboratory. We suggest a long term study over several years, in order to sample nests from seasons with a variety of thermal and climactic conditions.

### Hatchling size and mass

A negative correlation between incubation temperature and sea turtle hatchling size has been documented in all species of sea turtle studied (Hewavisenthi and Parmenter, 2001; Booth et al., 2004; Ischer et al., 2009; Mickelson and Downie, 2010; Booth and Evans, 2011; Read et al., 2012; Wood et al., 2014; Maulany et al., 2012a). Similarly, we found that hatchlings from nests with a T3dm above 34°C had smaller carapaces than hatchlings from nests with a T3dm below 34°C. This is likely due to warmer nests experiencing shorter incubation duration, meaning the hatchlings produced have less time to convert their yolk into tissue before hatching. Thus they are smaller in dimensions, but have a larger residual yolk compared to hatchling from cooler nests (Booth and Astill, 2001; Booth, 2006; Ischer et al., 2009). This likely explains why we found a difference in hatchling size but not mass, as the total mass of the hatching and yolk sac would be similar.

Previous studies following a similar relocation protocol have found no difference in size or between relocated and *in situ* nests (Pintus et al., 2009; Tuttle and Rostal, 2010). Another study found differences in the mass and width of hatchlings from relocated vs. *in situ* nests, but these differences were so small, they are unlikely to be biologically relevant (Türkozan and Yilmaz, 2007).

Larger hatchlings likely have a greater chance of survival, due to their ability to travel more quickly through the predator-rich beach and near-shore environments (Chu, 2008). Larger hatchlings also may be ignored by gape-limited predators (Bustard, 1972), however smaller hatchlings with larger yolk stores will have greater energy stores and will be able to travel further without feeding (Ischer et al., 2009). Thus there is a trade-off and the effect on fitness and survival will vary depending on predation pressure at rookeries.

### Terrestrial locomotor performance

Crawling speed has been negatively correlated with mean incubation temperature before in several field-based sea turtle studies (Chu, 2008; Ischer et al., 2009; Maulany et al., 2012a; Read et al., 2012; Booth et al., 2013; Wood et al., 2014). In *C. caretta* hatchlings this difference remained, even after the authors added hatchling size as a covariate (Chu, 2008; Read et al., 2012), suggesting that increased crawling speed is not solely due to the difference in size. Our study showed that hatchlings from nests with a T3dm below 34°C crawled more quickly than hatchlings from nests with a T3dm above 34°C, supporting the above trend. As in the other study, the difference remained even after controlling for the larger body size of hatchlings from cooler nests. This suggests that even though the time spent about 34°C in the late stages of incubation was not enough to be fatal, it may have had a detrimental effect on the physiology of the developing embryo. The embryos are almost fully developed at this stage of development (Miller, 1985), and high temperatures can cause uncoordinated movement in emergent hatchlings (Drake and Spotila, 2002), therefore the same mechanism may be occurring in pre-emergent hatchlings. Crawling speed affects the amount of time the hatchling spends on the beach, with longer on beach periods increasing the chance of terrestrial predation, or becoming dehydrated or overheated (Dial, 1987; Burgess et al., 2006; Janzen et al., 2007).

Despite differences in size, there was no difference in either self-righting propensity or time taken to self-right between the two groups. Two previous studies using the mean incubation temperature of relocated nests found that hatchlings from warmer nests were less likely to self-right within 10 seconds, and take longer to self-right than hatchlings from cooler nests (Read et al., 2012; Wood et al., 2014). The range of mean incubation temperatures were similar in all studies (27.9–32.6°C in this study and 29.6–32.2°C (Wood et al., 2014), 28.1–32.5°C (Read et al., 2012)), and so was the range of body sizes, so this suggests that self-righting ability depends on a wider range of temperatures that just the T3dm. Also, since turtle hatchlings self-right by flexing their head against the substrate (Booth et al., 2013), neck length may be important to self-righting ability, but has not been measured in self-righting studies. Another limitation of this study is the fact that each hatchling had to be tested individually and therefore hatchlings tested towards the end would be more fatigued than those tested at the beginning.

### Swimming performance

Swimming effort of sea turtle hatchlings generally decreases with time spent swimming, with the largest decreases occurring within the first two hours of entering the water (Pilcher and Enderby, 2001; Booth et al., 2004; Booth, 2009; Ischer et al., 2009; Booth and Evans, 2011; Pereira et al., 2011). Similarly, hatchlings in this study decreased their swimming effort in terms of thrust produced, stroke rate, proportion of time spent power-stroking and mean maximum thrust over the four hour testing period. Hatchlings are most vulnerable to predation in the shallow near-shore environment (Salmon and Wyneken, 1987; Gyuris, 1994) and by maximising their swimming effort at the beginning of their swim, they will move out of this zone rapidly. The swimming effort then decreases as the hatchlings fatigue.

Hatchlings from Mon Repos rookery must swim approximately 5–10 km offshore to reach the south-east Australian current, which then carries them to their juvenile feeding grounds (Walker, 1994). Hatchlings from nests with a T3dm below 34°C produced more thrust than hatchlings from nests with a T3dm above 34°C in the first twenty minutes of swimming. This period is likely the most important in terms of survival, as the near shore environment is the most predator-dense (Gyuris, 1994; Pilcher et al., 2000; Salmon et al., 2009). Although we did not measure swimming speed directly, hatchlings were of a uniform shape and would have a similar hydrodynamic resistance. Therefore thrust can be considered a proxy for swim speed (Booth and Evans, 2011). Previous work on sea and freshwater turtle hatchlings has suggested that hatchlings from a mid-range incubation temperature perform better in swimming trials than those from either high or low mean incubation temperatures (Chu, 2008; Booth et al., 2004; Burgess et al., 2006). Again, this might be due to adverse effects of extreme high or low temperatures on the physiology of the developing embryo. With impending global climate change, global air temperatures (and therefore nest temperatures) will increase over the next few years (Hays et al., 2003; Hays, 2008; Fuentes et al., 2011). This could mean a decrease in swimming performance of sea turtles.

### Conclusions

This study demonstrates that sub-lethal incubation temperatures late in incubation can have negative effects on both morphology and locomotor performance of marine turtle hatchlings. Sub-lethal temperatures can affect hatchling emergence, size, and crawling and swimming ability. As global temperatures rise, the proportion of nests experiencing extreme high temperatures is likely to increase, which may affect hatchling survival rates.
